# Treatment trends in allergic rhinitis and asthma: a British ENT survey

**DOI:** 10.1186/1472-6815-11-3

**Published:** 2011-04-11

**Authors:** Ravinder S Natt, Petros D Karkos, Davinia K Natt, Eva G Theochari, Apostolos Karkanevatos

**Affiliations:** 1 Department of Otolaryngology, Leighton Hospital, Crewe CW1 4QJ, UK; 2 Department of Otolaryngology, Royal Liverpool University Hospital, Liverpool L7 8XP, UK; 3 Department of General Medicine, Leighton Hospital, Crewe CW1 4QJ, UK; 4 Division of Primary Care, University of Liverpool, Liverpool L69 3BX, UK

**Keywords:** Allergic Rhinitis, Asthma, Survey, Questionnaire, Atopy

## Abstract

**Background:**

Allergic Rhinitis is a common Ear, Nose and Throat disorder. Asthma and Allergic Rhinitis are diseases with similar underlying mechanism and pathogenesis. The aim of this survey was to highlight current treatment trends for Allergic Rhinitis and Asthma.

**Method:**

A questionnaire was emailed to all registered consultant members of the British Association of Otorhinolaryngologists - Head and Neck Surgeons regarding the management of patients with Allergic Rhinitis and related disorders.

**Results:**

Survey response rate was 56%. The results indicate a various approach in the investigation and management of Allergic Rhinitis compatible with recommendations from the Allergic Rhinitis and Its Impact on Asthma guidelines in collaboration with the World Health Organisation.

**Conclusion:**

A combined management approach for patients with Allergic Rhinitis and concomitant Asthma may reduce medical treatment costs for these conditions and improve symptom control and quality of life.

## Background

The definition of Allergic Rhinitis (AR) was formulated by Hansel in 1929 [1]. It is a symptomatic nasal disorder caused by allergen exposure through an IgE-mediated immune response against allergens. AR can be subdivided into intermittent (symptoms <4 days per week or for <4 weeks) and persistent disease (symptoms >4 days per week or for >4 weeks) and is further characterized according to severity as mild or moderate/severe [2].

The nasal passage and paranasal sinuses are an integral part of the respiratory tract and patients may have rhinitis without sinusitis, but not sinusitis without rhinitis, hence the term rhinitis has been replaced in modern ENT literature by the more accurate term rhinosinusitis [3]. AR and Asthma are linked epidemiologically, pathologically, physiologically and therapeutically and can be considered as a manifestation of a single inflammatory airway syndrome [4]. Most patients with Asthma have rhinitis suggesting the concept of "one airway, one disease" [5]. AR is more prevalent than Asthma and a European population study was reported to have an AR prevalence rate of 25% [6]. AR usually precedes Asthma and can be considered as risk factor for the development of Asthma. Rhinitis exists in up to 80% of Asthma patients and frequently exacerbates Asthma and increases the risk of Asthma attacks, but the prevalence of Asthma in patients with rhinitis varies from 10 - 40% [7]. AR and Asthma are chronic respiratory diseases that cause major disability worldwide including impaired sleep, school, work and quality of life and are associated with substantial economic costs [8].

The aetiology of AR is multi-factorial and the diagnostic and therapeutic choices remain diverse. The aim of this questionnaire survey was to highlight the treatment trends in the management of AR amongst UK-based otolaryngologists.

## Methods

A questionnaire (Appendix 1) was emailed via ENT UK to the email addresses of all registered consultant members of the British Association of Otorhinolaryngologists - Head and Neck Surgeons (BAO-HNS). Survey recipients were asked 1) about their familiarity with the Allergic Rhinitis and Its Impact on Asthma (ARIA) guidelines in collaboration with the World Health Organisation (WHO), 2) about the type of investigations, treatment regimes and follow-up arrangements for AR patients and 3) about whether they give to patients advice leaflets on life style changes including education and allergen avoidance. All participants had the opportunity to answer anonymously.

## Results

**Figure 1:**
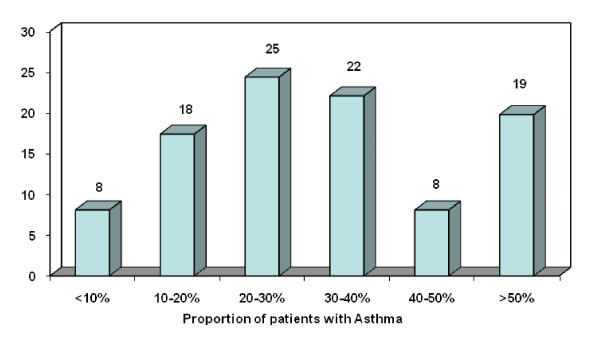
**Proportion of Allergic Rhinitis patients that have Asthma**.

A total of 551 questionnaires were emailed. There were 309 replies (56% response rate). All consultants were familiar with the association between AR and Asthma but only 63% of the respondents were familiar with the ARIA guidelines. Seventy seven (25%) of otolaryngologists managed AR patients of which a 20 - 30% proportion had associated symptoms or a diagnosis of Asthma (Figure 1).

**Figure 2:**
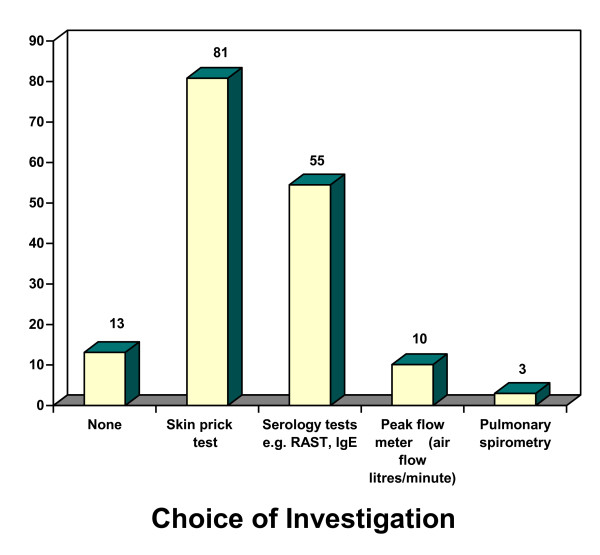
**Investigations for Allergic Rhinitis**.

The commonest investigation requested was skin prick testing (81% of respondents). Only nine consultants (3%) arranged pulmonary spirometry (Figure 2).

**Figure 3:**
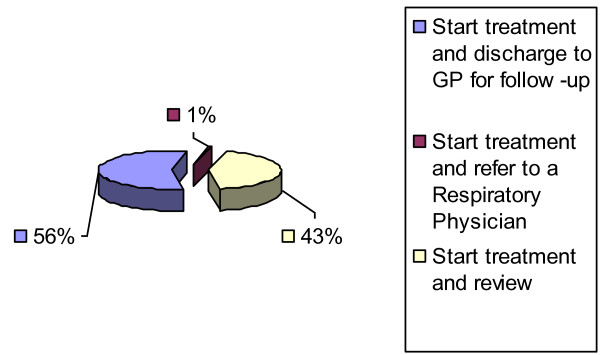
**Management Pathway for Allergic Rhinitis**.

Fifty six per cent of otolaryngologists preferred the management pathway of starting treatment and then discharging AR patients to General Practitioners (GPs) for further follow-up. However three (1%) consultants managed and subsequently referred AR patients to a respiratory physician (Figure 3).

**Figure 4:**
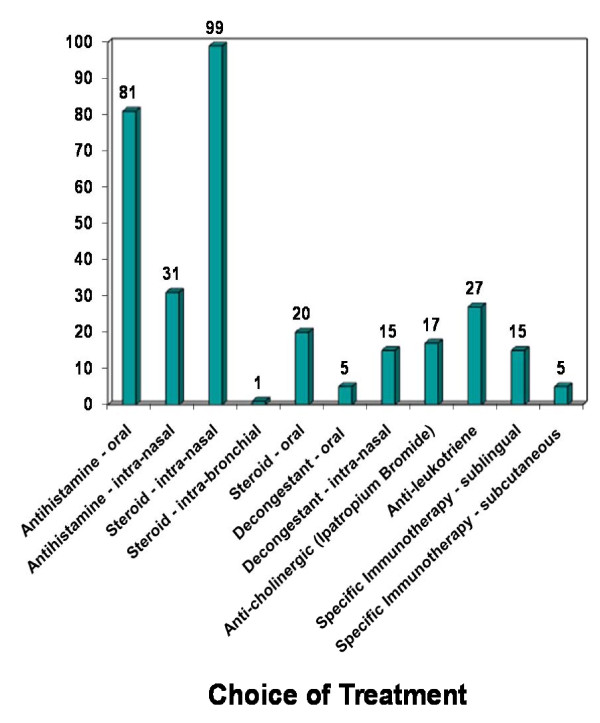
**Treatment for Allergic Rhinitis**.

In total two hundred and forty (78%) of otolaryngologists provided patient leaflets on life-style changes including education and practical tips on allergens avoidance .(Figure 4)

## Discussion

AR is a multi-factorial disease with a worldwide disabling effect on all individuals irrespective of age and ethnic background. In 2001 the ARIA in collaboration with the WHO workshop published guidelines for healthcare professionals in order to highlight the latest updates on the aetiology of AR emphasizing the association between AR and Asthma and proposing a management algorithm [2]. In 2008 a further update incorporated evidence based practice and the Grading of Recommendations Assessment, Development and Evaluation (GRADE) Working Group methodology with a focus on the prevention of allergic and chronic respiratory diseases [9].

Risk factors for AR include a combination of environmental and genetic interactions [10]. The diagnosis of AR is based upon a thorough history of allergic symptoms and diagnostic investigations. The European Academy of Allergiology and Clinical Immunology, the US Joint Council of Allergy and Asthma and the WHO recommend the use of skin prick testing for AR, which is preferred by the majority of UK otolaryngologists (81%) [11].

An overwhelming 99% of UK ENT consultants use intra-nasal steroids to treat AR. This trend is supported by several studies demonstrating that intra-nasal steroids are the most efficacious and cost effective first line treatment for AR [12,13].

Allergen specific immunotherapy for AR was first described in 1911 by Noon and involves the administration of a gradually increasing quantity of an allergen extract [14]. Patients are selected with demonstrated specific IgE antibodies to known allergens. In the USA the subcutaneous route is the only licensed route of administration. Interestingly 15% of UK otolaryngologists used sublingual and 5% used subcutaneous forms of immunotherapy. There is increasing evidence supporting the use of allergen immunotherapy in AR. Unlike pharmacotherapy, the clinical benefits are likely to be sustained for many years after discontinuation of treatment [15]. Wilson *et al *in a cochrane review showed sublingual immunotherapy to be a safe and effective option in managing AR [16]. Furthermore, Calderon *et al *in a recently published meta-analysis advocate the use of allergen injection immunotherapy as an effective treatment with a low risk of adverse effects in the management of AR [17]. However, further studies are required to evaluate significant differences between these two routes. Allergen specific immunotherapy is not recommended in patients with severe or uncontrolled Asthma because of risk of adverse bronchial reactions [18].

The importance of educating patients or carers with advice leaflets with information regarding AR including allergen avoidance cannot be over emphasised and was actively supported by 78% of responders of this survey. This enables individuals to be actively involved in the management of their disease resulting in an overall improvement in patient satisfaction, treatment compliance and outcomes [19]. Such practice is endorsed by the General Medical Council (GMC) through its guidance on good medical practice for doctors [20].

Interestingly, only 3% of otolaryngologists arranged pulmonary spirometry and 1% of all respondents referred AR patients to a respiratory physician for management of any associated lower respiratory tract pathology. Several studies though have demonstrated that AR patients with no underlying Asthma do have impaired pulmonary function and reversible airway obstruction [21,22]. In addition, studies have revealed that AR is linked to an increased use of Asthma related medical services and that the treatment of either AR or that of Asthma can alleviate the symptoms of the other and hence reduce the number of days off school and work and costs of utilising medical services loss of employment productivity [23,24]. Furthermore, greater awareness of the economic burden of AR would assist healthcare providers in establishing priorities for the allocation of their limited resources and ultimately ensure cost and clinical effective outcomes [25].

## Conclusion

This survey demonstrates as expected a diverse approach to the management of Allergic Rhinitis. An interesting finding of the survey is that despite good evidence on the relationship between Asthma and AR, most otolaryngologists often do no think "outside the specialty", for example they would rarely use spirometry as a diagnostic aid.

A combined treatment strategy of allergen avoidance, pharmacotherapy, immunotherapy and education applied to patients with AR and concomitant Asthma as recommended by the ARIA guidelines may reduce medical treatment costs and improve symptom control and quality of life.

## Conflicts of and Competing interests

The authors declare that they have no competing interests.

## Authors' contributions

RSN: Primary Author of manuscript. PDK: Assistant Author of manuscript and Editing. EGT: Contribution to Literature Search and Proof Reading. AK: Senior Clinician and contribution to Proof Reading and Editing. DKN: Assisted writing manuscript and designing questionnaire. All authors read and approved the final manuscript.

## APPENDIX 1: Allergic Rhinitis E-mail Questionnaire

Dear colleague,

Pease could you complete this survey with your responses and return it by clicking the SUBMIT button below. We thank you for your valuable time in completing this survey.

1. Are you familiar with the association between Allergic Rhinitis and Asthma?

• Yes

• No

2. Are you familiar with the Allergic Rhinitis and its impact on Asthma (ARIA) guidelines in collaboration with the World Health Organisation?

• Yes

• No

3. What proportion of your patients with Allergic Rhinitis have got symptoms or diagnosis of Asthma?

• <10%

• 10 - 20%

• 20 - 30%

• 30 - 40%

• 40 - 50%

• >50%

4. If you suspect Allergic Rhinitis what investigations would you consider (you may tick one, or more of the following)?

• None

• Skin prick test to known allergens

• Serology tests e.g. RAST, IgE

• Peak flow meter (air flow litres/minute)

• Pulmonary spirometry

5. If you suspect Allergic Rhinitis in a patient which of the following management plans would you consider?

• Start treatment and review

• Start treatment and discharge patient to GP for follow-up

• Start treatment and refer to a Respiratory Physician

6. If you suspect Allergic Rhinitis which of the following would you use for treatment (you may tick one, or more of the following)?

• Antihistamine - oral

• Antihistamine - intra-nasal

• Steroid - intra-nasal

• Steroid - intra-bronchial

• Steroid - oral

• Decongestant - oral

• Decongestant - intra-nasal

• Anti-cholinergic (Ipatropium Bromide)

• Anti-leukotriene

• Specific Immunotherapy - sublingual

• Specific Immunotherapy - subcutaneous

7. How long do you prescribe nasal sprays for

• 3 months

• 6 months

• >6 months

8. What is the frequency of nasal spray administration do you prescribe (you may tick one, or more from the following)?

• Two puffs once a day

• Two puffs twice a day

• Other (please specify)

9. Do you provide leaflets for patients about life-style changes including education and avoidance of allergen(s)?

• Yes

• No

## Pre-publication history

The pre-publication history for this paper can be accessed here:

http://www.biomedcentral.com/1472-6815/11/3/prepub
